# Genome Re-Sequencing and Functional Analysis Reveal an α-1,3-Glucosyltransferase Conferring Metalaxyl Resistance in *Phytophthora sojae*

**DOI:** 10.3390/jof12070479

**Published:** 2026-06-30

**Authors:** Jian Gao, Xiong Zhang, Peilin Wang, Shaocheng Chen

**Affiliations:** 1 Department of Plant Pathology, Southwest University, Chongqing 400715, China; 2 Department of Plant Pathology, Nanjing Agricultural University, Nanjing 210095, China; 3 Management Center of Yintiaoling National Nature Reserve, Chongqing 405800, China; 4 Department of Biological and Chemical Engineering, Chongqing University of Education, Chongqing 400065, China; 5 Chongqing Field Scientific Observation and Research Station for Authentic Traditional Chinese Medicine in the Three Gorges Reservoir Area, Chongqing 400065, China

**Keywords:** *Phytophthora sojae*, genome re-sequencing, *PsALG8*, metalaxyl resistance, mycelial growth

## Abstract

*Phytophthora* and allied oomycete pathogens pose a perennial challenge to global food security through their devastating impact on crop systems. While metalaxyl has demonstrated remarkable efficacy in controlling *Phytophthora* diseases since its introduction decades ago, the persistent emergence of metalaxyl-resistant strains has severely compromised its field efficacy. Elucidating the genetic determinants underlying resistance mechanisms is critical to developing surveillance strategies and sustainable countermeasures against evolving oomycete resistance. Through experimental evolution, we generated six metalaxyl-resistant *Phytophthora sojae* mutants exhibiting extreme resistance levels (resistance factor > 2000). Comparative whole-genome re-sequencing of resistant mutants versus the wild-type parental strain identified 64 candidate genes containing conserved nonsynonymous mutations across all resistant lineages. Among these, *PsALG8*, encoding a putative alpha-1,3-glucosyltransferase, was identified as the primary determinant, carrying a recurrent homozygous missense mutation across all resistant lineages. CRISPR/Cas9-mediated knockout of *PsALG8* in both wild-type and resistant backgrounds significantly reduced metalaxyl tolerance (*p* < 0.01), confirming its functional involvement in resistance modulation. These results suggest that *PsALG8* is associated with metalaxyl sensitivity and mycelial growth in *P. sojae* under laboratory conditions. The conservation of ALG8 homologs suggests that *PsALG8* may have a conserved cellular function related to protein glycosylation across eukaryotes. Although this glucosyltransferase is universally conserved among oomycete species, whether its association with metalaxyl sensitivity constitutes a shared resistance adaptation pathway still requires extensive functional validation in diverse *Phytophthora* pathogens, which may offer insights into future fungicide resistance management strategies in *P. sojae*.

## 1. Introduction

Oomycetes, though morphologically similar to fungi, constitute a distinct group of stramenopilan eukaryotes phylogenetically aligned with diatoms and brown algae [1]. These economically devastating pathogens inflict significant economic losses across agricultural systems through infection of vital crop plants, livestock, and occasionally humans [2,3]. The most notorious plant-infecting oomycete pathogens belong to the genus *Phytophthora*, which includes nearly 200 species [4]. Among these, *P. infestans*, the causal agent of the Great Irish Potato Famine [5], and *P. sojae*, a primary cause of soybean root and stem rot responsible for approximately $2 billion in annual losses worldwide [6], exemplify the threat to herbaceous crops. Furthermore, *Phytophthora* species represent a persistent and escalating threat to high-value woody crops, such as walnut (*Juglans* spp.), where diseases like root rot and collar rot often lead to severe decline or mortality. Current disease management paradigms combine host resistance deployment with targeted oomyceticides (specific antifungal agents with oomycete efficacy). However, the evolutionary plasticity of oomycete pathogenicity determinants frequently enables rapid breakdown of genetic resistance in commercial cultivars [7]. This evolutionary arms race has consequently elevated chemical interventions to cornerstone status in contemporary oomycete disease control despite growing concerns about resistance development and environmental impacts [8,9,10].

Chemical oomyceticides have been used to control oomycete diseases since the 19th century [11]. In the last few decades, chemicals that exhibit specificity to oomycetes have been developed. An example of these oomyceticides is metalaxyl (methyl N-(methoxyacetyl)-N-(2, 6-xylyl)-DL-alaninate). It belongs to the phenylamides group, which includes metalaxyl-M, furalaxyl, benalaxyl, benalaxyl-M, ofurace, and oxadixyl as well (FRAC Code List 2024). Metalaxyl was first introduced into the market in the late 1970s. It displays excellent performance in controlling all oomycete pathogens on many crops worldwide, including *Phytophthora*, *Pythium*, and downy mildews [12]. For example, treating seeds with metalaxyl has been a very effective method for managing seed-borne diseases of many crops [13]. However, metalaxyl has a high intrinsic resistance risk, according to the Fungicide Resistance Action Committee (FRAC). Metalaxyl-resistant isolates of *Pseudoperonospora cubensis* were detected in Israel only 2 years after the introduction of metalaxyl [14]. Metalaxyl resistance in *Plasmopara viticola* has also been detected in vineyards in Europe since 1981 [9]. With long-term intense use, metalaxyl resistance is becoming increasingly severe in many oomycete species. Taking *P. infestans* as an example, it was reported that all collected strains in Peru and over 90% in China showed resistance to metalaxyl [15]. Metalaxyl is also one of the most widely used oomyceticide to control *Phytophthora* root rot of soybean. Interestingly, no *P. sojae* strains have been found to be resistant or even insensitive to metalaxyl in the field so far, although a potential risk may occur after a long-term application in soybean fields [16,17,18]. While metalaxyl is widely used to manage *Phytophthora* diseases in soybeans, the emergence of resistance in walnut-infecting *Phytophthora* and other pathogens highlights an urgent, cross-species need to understand the underlying molecular mechanisms.

In the past three decades, intensive progress has been achieved in understanding the mode of action of metalaxyl in *Phytophthora*. Biochemical studies on *P. megasperma* and *P. infestans* found that metalaxyl inhibits the synthesis of ribosomal RNA, suggesting that it may target RNA polymerase I [19]. Randall reported a single nucleotide polymorphism (SNP) in the large subunit of RNA polymerase I (RPA190) that was strongly associated with insensitivity to metalaxyl, and this allele was sufficient to confer insensitivity when transformed into the sensitive isolates [11]. This study showed that SNP T1145A (the corresponding amino acid is F382Y) of RPA190 was 86% associated with metalaxyl insensitivity in *P. infestans*. However, a study reported that resistant isolates lacking the T1145A SNP were observed in *P. infestans*, suggesting that other unknown genes might be involved in metalaxyl resistance [12]. However, interestingly, Chen reported mutations (including V1476G, P980S, and F382Y) in RPA190 were positively correlated with metalaxyl resistance in *P. infestans* [15]. A conserved domain RPOLA-N of the RPA190 gene of *P. capsici* was reported to involve in the regulation of *P. capsici* resistance to metalaxyl. Obviously, the molecular mechanisms that contribute to metalaxyl resistance are still inconclusive. It was previously suggested that metalaxyl resistance might be determined by multiple loci [20]. In 2015, Childers et al. found that several *P. infestans* genes might contribute to metalaxyl resistance [21]. Additionally, GC-MS metabolite profiling analyses suggested that an increase in membrane rigidity might be associated with metalaxyl resistance in *P. infestans* [22].

Resistance risk assessment is essential to prevent or delay the development of fungicide resistance [23]. Thus, the objectives of the present study aimed to determine the risk of *P. sojae* isolates developing resistance to metalaxyl, and to investigate the potential resistance mechanisms. We obtained laboratory-induced metalaxyl-resistant mutants of *P. sojae*, and took advantage of the genome re-sequencing approach to identify potential genes that are associated with metalaxyl resistance.

## 2. Materials and Methods

### 2.1. Generation of Laboratory-Induced Metalaxyl-Resistant P. sojae Mutants

The *P. sojae* parental isolate P7076 was used as the wild-type isolate to generate metalaxyl-resistant mutants. The metalaxyl-sensitive parental isolate P7076 was originally obtained from the wild-type -*P. sojae* isolate P7076, which was kindly provided by Professor Yuanchao Wang, Nanjing Agricultural University, and maintained on V8 agar medium at 25°. For details of the strain source, please refer to the website https://www.apsnet.org/publications/mpmi/backissues/Documents/1994Abstracts/Microbe07-780.htm (accessed on 10 December 2019). The laboratory-induced resistant strains were generated only from the parental isolate P7076. Additional field isolates of *P. sojae* were used solely for PsALG8 sequence comparison and were not used in the resistance induction experiment. Fresh mycelial plugs from 5-day-old colonies were transferred to V8 plates containing 5 μg/mL metalaxyl. After dark incubation at 25 °C for 15–30 days, the fastest-growing portion of each colony was transferred to fresh V8 plates amended with increasing concentrations of metalaxyl (5, 10, and 100 μg/mL). Mycelial plugs from the actively growing colony margin were transferred to fresh medium every 5–7 days during this selection process. Finally, after 30 times of subculture, the surviving colonies were transferred onto V8 plates containing 150 μg/mL metalaxyl to maintain the acquired resistance. Colony diameter was measured as an indicator of mycelial growth. The EC50 values of metalaxyl-resistant strains were then estimated by measuring colony diameter on V8 plates containing 500, 750, 1000, 1250, 1500, 1750, and 2000 μg/mL of metalaxyl. The resistance factor (RF) for each strain was calculated by dividing the EC50 of the mutant by that of the wild-type parental isolate.

### 2.2. Sensitivity of P. sojae Isolates to Metalaxyl

A mycelial growth inhibition assay was used to evaluate the sensitivity of *P. sojae* isolates to metalaxyl [16]. Briefly, 100 μg/mL stock solution of metalaxyl was produced by dissolving metalaxyl in dimethyl sulfoxide (DMSO) and kept at 4 °C in darkness until use. Mycelial plugs (5 mm in diameter) were taken from the actively expanding 5-day-old *P. sojae* colonies and transferred to the fresh V8 plates amended with metalaxyl at the following concentrations of 0.02, 0.05, 0.10, 0.20, 0.40, 0.60, and 0.80 μg/mL. The colony diameter was measured in two perpendicular directions after 5 to 7 days of darkness incubation at 25 °C when the control colonies (without metalaxyl) were at least 6 cm in diameter. Three technical replicate plates were set for each treatment, and the entire experiment was performed independently three times. The growth inhibition ratio was calculated according to the formula: [(colony diameter of the control − colony diameter of treatment)/(colony diameter of the control − 5 mm) × 100].

### 2.3. Mycelial Growth, DNA Extraction, and Whole-Genome Sequencing

The wild-type parental and all metalaxyl-resistant *P. sojae* isolates in this study were routinely grown on oomyceticide-free V8 agar plates at 25 °C, as described previously [24]. The mycelia were harvested after 6 days of dark incubation.

Genomic DNA was extracted according to the CTAB (hexadecyl trimethyl ammonium bromide) method modified by Cheeseman [25]. The purified genomic DNA was fragmented to an average insert size of approximately 350 bp. DNA quantification was performed using the Qubit 2.0 Fluorometer kit. Library construction was carried out following the standard Illumina protocol, including end repair, A-tailing, adaptor ligation, and PCR enrichment. Library quality was assessed using the Agilent 2100 Bioanalyzer (Agilent Technologies, Santa Clara, CA, USA). All samples were sequenced using the Illumina HiSeq X-ten platform (Illumina, San Diego, CA, USA) at a 300 base-pair (bp) of paired-end read length. All the sequencing data were deposited to the NCBI Sequence Read Archive (SRA) under the BioprojectPRJNA639683.

### 2.4. Reads Mapping and Variants Calling

Raw reads were filtered using the clean_adapter and clean_lowqual website (https://github.com/fanagislab/common_use) (accessed on 25 September 2019). Clean reads were then aligned to *P. sojae* reference genome v3.0 downloaded from the NCBI database (accession number GCA_009848525.1) using the Burrows Wheeler Transform Alignment (BWA) software package v0.7.5a [26,27]. For each sample, duplicate reads were removed from alignments using Samtools software package v1.8 [28]. The mpileup function in Samtools was used to generate mpileup files for each sample. Bcftools and vcftools were used to identify SNPs in individual samples [29]. The following criteria were applied during SNP filtering: (i) the Phred quality score of base sequencing and score of read mapping higher than 60; (ii) minimum coverage higher than 10; (iii) allele frequency ≥80% for homozygous SNPs; and (iv) allele frequency between 20% and 80% for heterozygous SNPs. Whole-genome re-sequencing data generated in this study have been deposited in the NCBI Sequence Read Archive under BioProject accession number PRJNA639683. The accession numbers for individual samples are listed in Supplementary Table S4. Other data supporting the findings of this study are available within the article and in the Supplementary Materials.

### 2.5. Sequence Analysis of PsALG8

The gene was designated *PsALG8*, representing the *P. sojae* homolog of *ALG8*, a predicted α-1,3-glucosyltransferase involved in N-linked glycosylation. *PsALG8* (Ensembl ID: EGZ16653) from *P. sojae* was amplified from genomic DNA and then cloned into pBluescript II KS+ plasmids using an In-Fusion HD Cloning Kit (Clontech, Mountain View, CA, USA). PCR products were sent to Sango Biotech for sequencing. The used primers are listed in Table S1. The protein function was annotated using Uniprot (https://www.uniprot.org/) (accessed on 12 September 2024). Conserved domain was detected using Pfam (http://pfam.xfam.org/search) (accessed on 15 September 2019). Transmembrane region was predicted using TMHMM (http://www.cbs.dtu.dk/services/TMHMM/) (accessed on 14 September 2024). ALG8 homologs were identified from *P. sojae* and other oomycete species using BLASTP (https://blast.ncbi.nlm.nih.gov/Blast.cgi?PROGRAM=blastp&PAGE_TYPE=BlastSearch&LINK_LOC=blasthome, accessed on 13 June 2026). The accession numbers of all sequences used are listed in Supplementary Table S4. Amino acid sequences were aligned using MAFFT; multiple sequence alignments were also performed using MUSCLE v3.8.31 [30]. Homologs of PsALG8 in additional *P. sojae* isolates were retrieved from a previous report [31], while *PsALG8* homologs from other oomycete species were obtained from FungiDB (http://fungidb.org/) (accessed on 15 October 2019). Phylogenetic reconstruction was performed using the maximum likelihood method in PhyML v3.0. The best-fit substitution model was selected by ModelFinder, and branch support was assessed with 1000 bootstrap replicates [32]. Gene Ontology (GO) annotation was carried out with Blast2GO [33]. GO categories were analyzed using Web Gene Ontology Annotation Plotting (WEGO) tool [34].

### 2.6. Plasmid Construction and CRISPR/Cas9 Editing for PsALG8 Knockout

The sgRNA of *PsALG8* was predicted via EuPaGDT (http://grna.ctegd.uga.edu/) (accessed on 10 October 2019) with secondary structure and off-target assessment, and sequences spanning restriction sites were preferred for convenient mutant identification. Verified sgRNA fragments were seamlessly cloned into Fang’s pYF515 vector [35] after annealing and ligation, then validated by colony PCR and sequencing. For knockout donor construction, the NPTII-containing replacement cassette flanked by 1.0 kb upstream and downstream homologous arms of *PsALG8* was inserted into pBluescript II KS+ using the In-Fusion HD Cloning Kit (Clontech, Mountain View, CA, United States).

*PsALG8* knockout was achieved through optimized PEG-mediated protoplast co-transformation following the reported CRISPR/Cas9 system [35,36]. Transformants were screened on G418-containing medium and further confirmed by target-specific PCR and Sanger sequencing. All primer sequences are summarized in Table S1, and Figure S2 depicts the complete gene replacement strategy.

### 2.7. Pathogenicity Assay of ΔPsALG8 Mutants

For plant inoculation, wild-type parental isolate P7076, knockout lines TP-6/TP-13, and non-edited control TP-2 were incubated in 10% V8 broth in darkness for 2 days. Zoospores were then induced by washing with sterilized distilled water. The soybean cultivar Hefeng 47, which is compatible with *P. sojae* strain, was planted in plastic pots containing vermiculite and grown in darkness for 4 days. Etiolated soybean seedlings were inoculated with 100 zoospores of P7076 and transformants. The etiolated seedlings were then maintained in 80% humidity and darkness at 25 °C. Pathogenicity symptoms were evaluated after 2 days [35].

## 3. Results

### 3.1. Generation of Metalaxyl-Resistant Isolates

**Figure 1:**
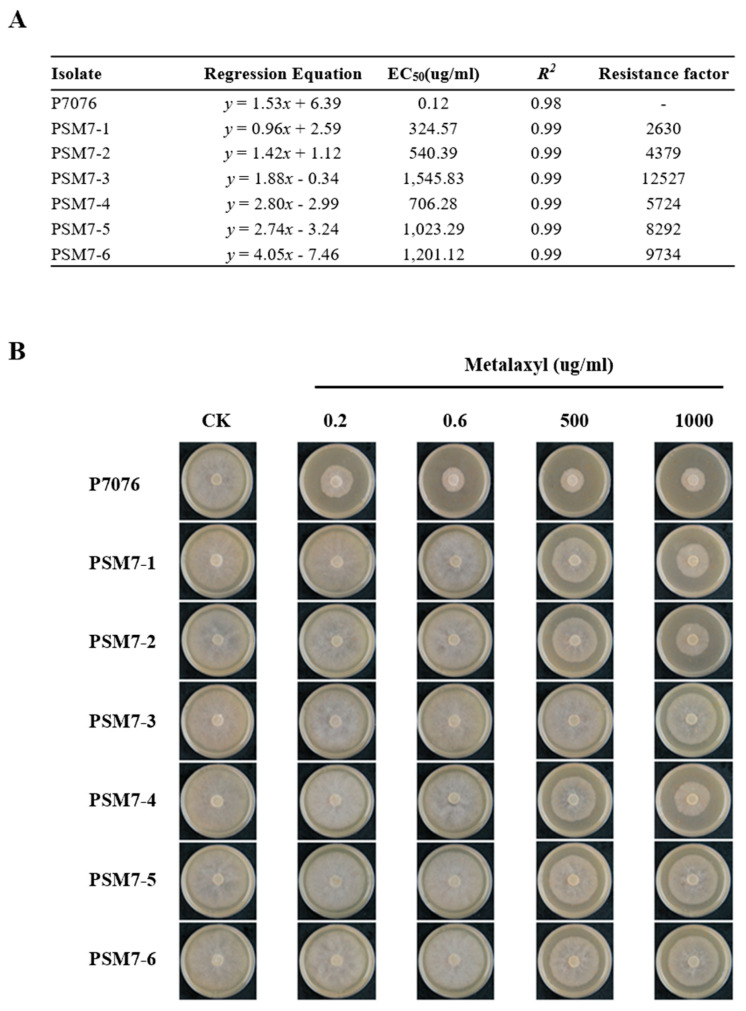
Generation of metalaxyl-resistant mutants. (**A**) The sensitivity of the parental isolates and mutants to metalaxyl. Resistance factor indicates EC50 of the resistant mutant/EC50 of the wild-type isolate P7076. (**B**) Mycelia growth of *P. sojae* isolates on metalaxyl-amended medium. All isolates were grown at 25 °C for 7 days on V8 media with the indicated concentrations of metalaxyl.

The inhibitory effect of metalaxyl on the mycelial growth of the wild-type *P. sojae* isolate P7076 was evaluated, and the EC50 was 0.12 μg/mL (Figure 1A), which was consistent with the results of previous studies [16,18], indicating that wild-type isolate was sensitive to metalaxyl. Six resistant mutants (designated as PSM7-1, PSM7-2, PSM7-3, PSM7-4, PSM7-5, and PSM7-6) were successfully obtained after 30 successive transfers of the wild-type parental isolate P7076 on metalaxyl-amended medium (Figure 1A). EC50 values of these resistant mutants ranged from 324.57 μg/mL to 1545.83 μg/mL, and the resistance factors (RFs) were all >2000, indicating that these mutants were highly resistant to metalaxyl. As shown in Figure 1B, the mycelia growth of P7076 was significantly reduced by 0.2, 0.6, 500, and 1000 μg/mL of metalaxyl in comparison with that without metalaxyl, whereas the mycelia growth of the six resistant mutants with 0.2 and 0.6 μg/mL of metalaxyl did not differ significantly from those of the control without metalaxyl. The mutants also displayed significantly greater growth than the wild-type isolate with 500 and 1000 μg/mL of metalaxyl (Figure 1B). Notably, the resistant mutants exhibited similar growth patterns with P7076 on the metalaxyl-free medium, suggesting that the mutants were not impaired in normal mycelial growth.

### 3.2. Analysis of Whole-Genome Re-Sequencing

To investigate the potential resistance mechanisms in the six resistant mutants, whole-genome re-sequencing was performed using Illumina Hiseq X-ten PE150. A total of 29.11 Gb pair-end (PE) data were generated with sequencing depth of 47.31X to 59.87X coverage (Table S2). After removing low-quality reads, approximately 28.17 Gb high-quality PE reads were mapped to the reference genome V3.0 of *P. sojae* [26]. When analyzed individually, the number of high-quality PE reads per resistant isolate ranged from 4.14 to 5.29 Gb, of which 92.46 to 96.17% were aligned with the reference genome, yielding 3.91 to 4.95 Gb high-quality PE reads (Table S2).

Compared with the parental isolate P7076, a total of 4242 single nucleotide polymorphisms (SNPs) were identified in the six resistant mutants based on a read support criterion of at least five reads, corresponding to an SNP frequency of 0.05 per 1 kb. PSM7-2 has the lowest frequency of SNPs (2542 SNPs), whereas PSM7-6 has the highest frequency of SNPs (2706 SNPs). Among these SNPs, the base changes were mainly G to A and C to T transitions, and the proportion was 25.22% and 23.29%, respectively (Figure S1A). The proportions of SNPs identified in coding sequences (CDS), introns and intergenic regions were 29.35%, 7.38% and 63.27%, respectively (Figure S1B). In total, 619 synonymous and 651 nonsynonymous SNPs were detected in the coding sequences. Among the nonsynonymous SNPs, 25 SNPs were found to introduce stop codons in five genes, whereas 6 SNPs disrupt stop codons in 23 genes.

### 3.3. Identification of Candidate Genes Associated with Metalaxyl Resistance

**Figure 2:**
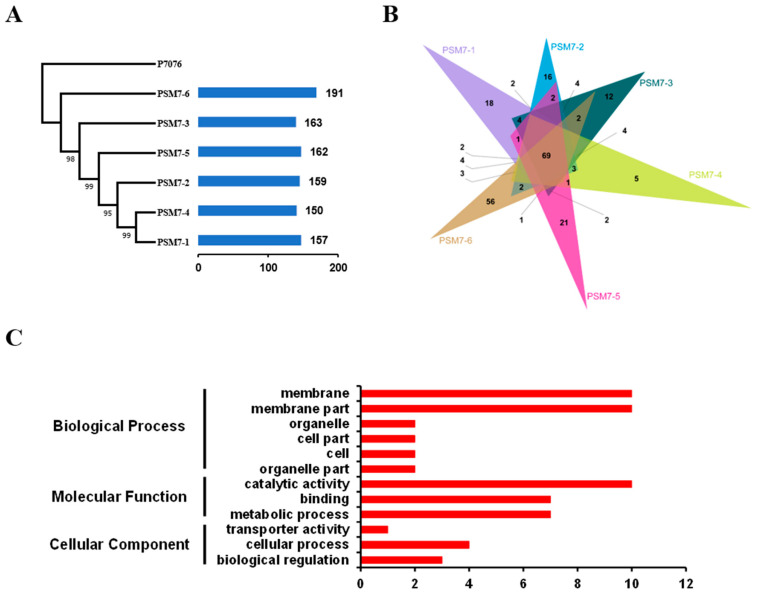
Identification of candidate genes associated with metalaxyl resistance. (**A**) Numbers of genes associated with amino acid substitutions in the six metalaxyl-resistant isolates. The maximum likelihood tree was constructed using PhyML v3.0 with 1000 bootstrap replicates based on 4242 SNPs. (**B**) Venn diagram of genes associated with amino acid substitutions in the six metalaxyl-resistant isolates. (**C**) Histogram of Gene Ontology (GO) categories of 69 candidate genes associated with metalaxyl resistance. Results are summarized in three main categories: biological process, cellular component, and molecular function. The x-axis indicates the number of genes in a category. The y-axis indicates GO categories.

Phylogenetic analysis based on the 4242 detected SNPs clearly distinguished the six metalaxyl-resistant mutants from their wild-type parental isolate P7076 (Figure 2A). Because the branch support separating P7076 and the laboratory-induced resistant strains was low, this analysis was interpreted only as a descriptive clustering result and not as strong evidence for phylogenetic divergence. Each mutant contained 150–191 mutated genes, ranging from 150 in PSM7-4 to 191 in PSM7-6. The absence of a non-selected P7076 passage control prevents us from determining which SNPs may have arisen from repeated subculturing alone. Considering that all resistant mutants originated from P7076 and point mutation serves as the predominant resistance-forming mechanism in oomycetes [11,37,38,39,40], we screened genes carrying amino acid substitutions and narrowed down the list to 324 candidate genes. Among them, 69 genes possessed consistent point mutations across all six resistant strains (Figure 2B), whereas other mutated genes were shared by 2–5 mutants or existed exclusively in a single isolate.

GO annotation showed that 21 out of the 69 genes were divided into 12 GO categories with three main categories (cellular component, molecular function, and biological process) (Figure 2C). For the cellular component category, the main subsets were member and membrane part, while the main subsets in the molecular function category were catalytic activity, binding, and metabolic process. Under the category of biological process, the main subsets were cellular process, biological regulation, and transporter activity.

### 3.4. Metalaxyl-Resistant Mutants Carried a Mutation of V422L in an Alpha-1,3-Glucosyltransferase

Previous studies reported that metalaxyl might target RNA polymerase I and that mutations in the largest subunit of RNA polymerase I (RPA190) were associated with metalaxyl resistance in *P. infestans* [11,15]. However, no SNPs were detected in any of the RNA polymerase I subunits. Therefore, we further analyzed all the SNPs in the above 69 genes and found all the SNPs were heterozygous in at least two metalaxyl-resistant isolates (Table S3). We focused exclusively on SNPs located within genes; therefore, Table S3 presents only those SNPs that fall within genic regions. Out of the 69 gene set, only EGZ16653 has the most frequently detected homozygous point mutation G1264C. We detected the G1264C mutation on EGZ16653 in all metalaxyl-resistant isolates (homozygous in PSM7-1, PSM7-2, PSM7-3, and PSM7-4; heterozygous in PSM7-5 and PSM7-6). Thus, we considered this gene as the most promising target for associating with metalaxyl resistance in our study.

**Figure 3:**
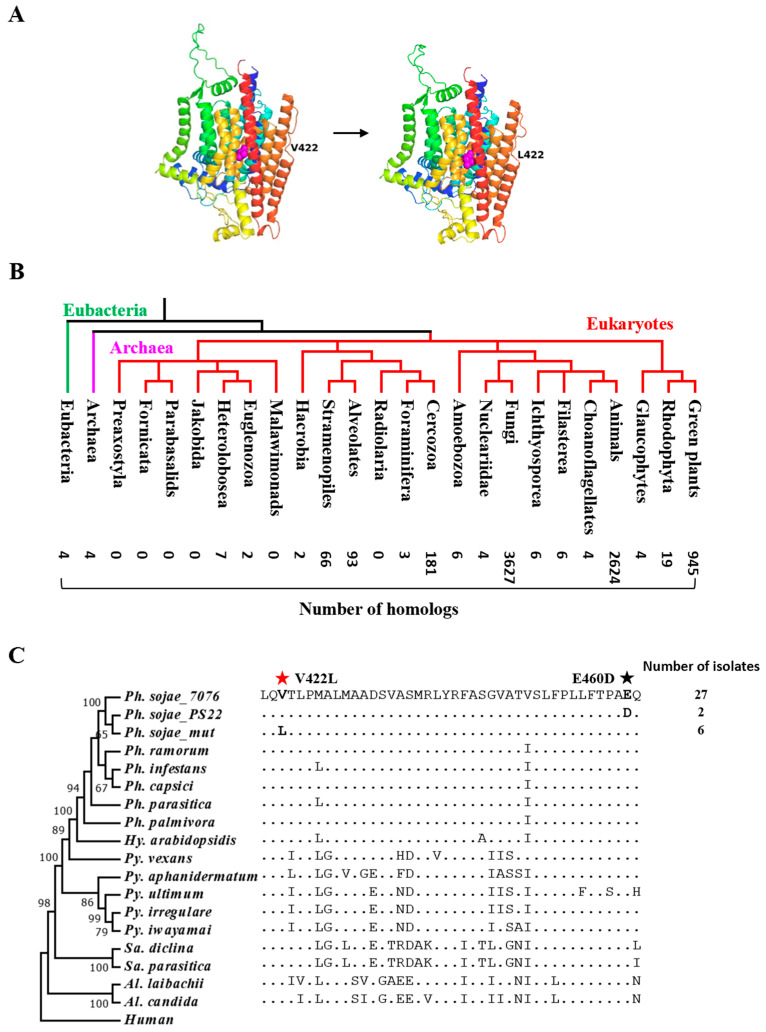
Metalaxyl-resistant mutants carry a mutation of V422L in PsALG8. (**A**) Predicted three-dimensional structure of PsALG8. (Mutant sites are marked in magenta.) (**B**) The distribution of ALG8 homologs in the tree of life. The top panel depicts phylogenetic relationships based on the tree of life phylogeny (http://tolweb.org/) (accessed on 10 October 2019). The bottom panel shows the numbers of ALG8 homologs identified in each lineage. (**C**) Polymorphism of V422L from 29 field *P. sojae* isolates, six metalaxyl-resistant *P. sojae* isolates, and 15 other oomycetes. V422L substitution was indicated by a red pentagram. E460D substitution was indicated by a black pentagram.

EGZ16653 encodes a protein of 575 amino acids with 42.1% identity to the alpha-1,3-glucosyltransferase (ALG8, GenBank accession: NP_076984.2). We named this gene PsALG8. The predicted three-dimensional structure of PsALG8 was generated using AlphaFold3 and visualized using PyMOL 2.6. Similar to the human ALG8 protein [41], the predicted structure of PsALG8 included ten transmembrane domains and an Alg6_Alg8 domain (Figure 3A). The V422L substitution caused by G1264C occurred in the Alg6_Alg8 domain and near the ninth transmembrane segment. The effect of the V422L substitution was examined by comparing the predicted structures of the wild-type and mutant PsALG8 proteins. (Figure 3A). ALG8 was highly conserved among eubacteria, archaea, and most of eukaryotes (Figure 3B) [42]. To analyze the evolution of ALG8 genes in oomycetes, various ALG8s were derived from available oomycetes genomes. Our results showed that ALG8 was a single-copy gene in all the detected oomycetes (Figure 3C). Multiple sequence alignments of ALG8s from 29 field *P. sojae* isolates, six metalaxyl-resistant *P. sojae* isolates, and 15 other oomycete species revealed that the valine at position 422 was highly conserved (Figure 3C), suggesting V422L substitution of ALG8 does not exist naturally in oomycetes. These results indicate that the molecular mechanism underlying metalaxyl tolerance in *P. sojae* may differ from that in *P. infestans*, and the specific V422L substitution in PsALG8 is associated with laboratory-induced metalaxyl resistance in *P. sojae*. A representative resistant strain carrying the PsALG8 V422L substitution was included in the phylogenetic analysis. The purpose of this analysis was to compare PsALG8 with ALG8 homologs from other oomycetes rather than to infer relationships among all laboratory-induced resistant strains.

### 3.5. Loss of PsALG8 Increased Sensitivity of P. sojae to Metalaxyl

**Figure 4:**
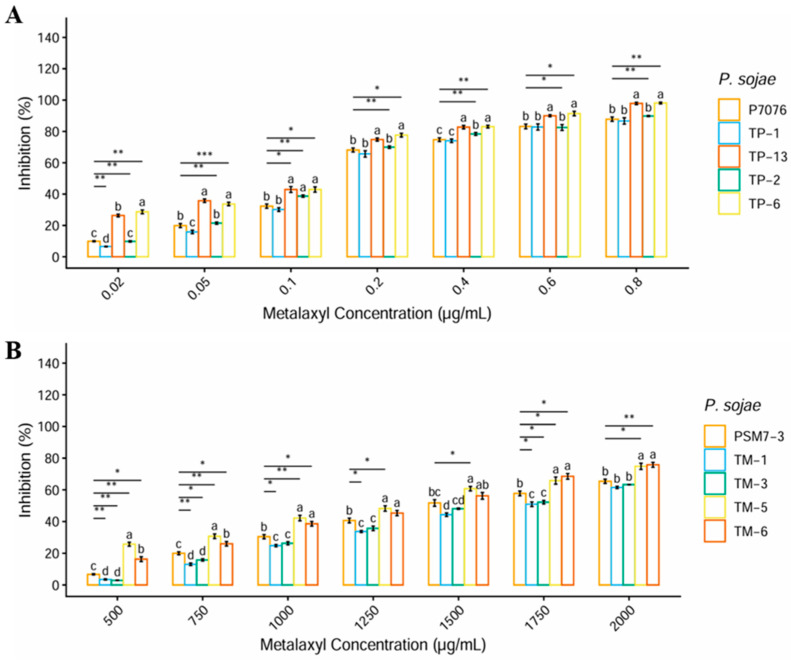
Colony diameter measurement of *PsALG8* knockout transformants to assess mycelial growth inhibition. (**A**) Wild-type P7076 background: knockout lines TP-6/TP-13 and non-edited control TP-2/TP-1under gradient metalaxyl treatments. (**B**) Laboratory-induced metalaxyl-resistant mutant PSM7-3 background: knockout lines TM-5/TM-6 and non-edited control TM-1/TM-3. All cultures incubated on V8 medium at 25 °C for 7 d. Different lowercase letters indicate significant differences at *p* < 0.05 within identical drug concentration. * indicates statistically significant difference at *p* < 0.05; ** indicates statistically significant difference at *p* < 0.01; *** indicates statistically significant difference at *p* < 0.001.

To examine the effect of PsALG8 on metalaxyl sensitivity, we attempted to knock out the PsALG8 gene using the reported CRISPR/Cas9 system [35]. Homozygous knockouts were successfully generated in the metalaxyl-sensitive isolate P7076 (designated as TP-6 and TP-13) and the resistant mutant PSM7-3 (designated as TM-5 and TM-6) (Figure S2). Firstly, we assessed the metalaxyl sensitivity of the four knockouts. The inhibition of 0.02, 0.05, 0.10, and 0.20 μg/mL metalaxyl on the mycelial growth of TP-6 and TP-13 were all significantly stronger than that on the wild-type isolate P7076 and the non-knocked isolates (TP-1 and TP-2) (Figure 4A). Similar results were also observed in TM-5 and TM-6, which were the PsALG8 knockouts of the resistant mutant PSM7-3. At all tested concentrations, the inhibitory effects of metalaxyl on mycelial growth of TM-5 and TM-6 were significantly stronger than that of their parent PSM7-3 (Figure 4B). These results indicated that deletion of PsALG8 increased the sensitivity of *P. sojae* to metalaxyl, suggesting that PsALG8 contributes to metalaxyl tolerance. However, additional mutations likely contribute to the high resistance phenotype.

### 3.6. PsALG8 Deletion Affected the Mycelia Growth of P. sojae

**Figure 5:**
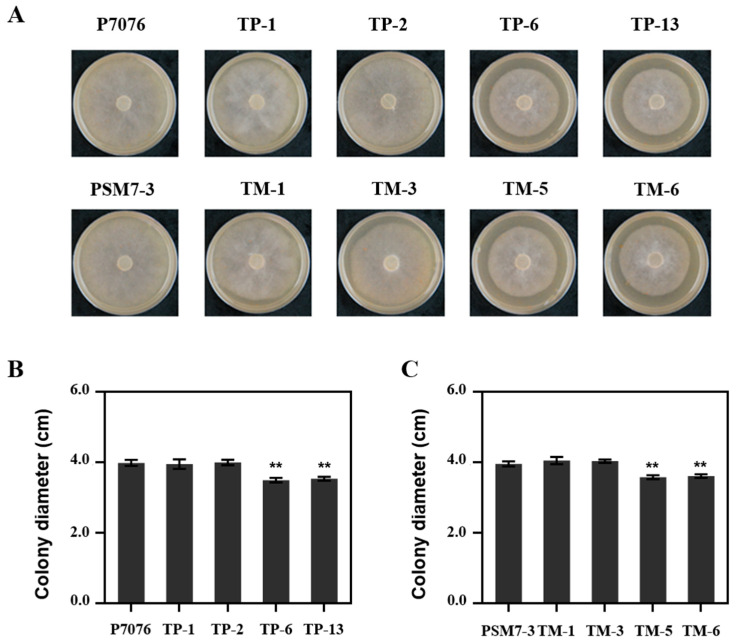
Colony diameter of *PsALG8* knockout transformants. (**A**) Colony morphology of tested strains cultured on V8 agar at 25 °C for 7 days. The *P. sojae* parental isolate P7076 and laboratory-induced metalaxyl-resistant strain PSM7-3 served as wild-type controls. TP-1/TP-2 and TM-1/TM-3 are non-edited control transformants; TP-6/TP-13 and TM-5/TM-6 are PsALG8-knockout transformants. (**B**) Colony diameters of the parental isolate P7076, non-edited control transformants (TP-1/TP-2), and PsALG8 knockout transformants (TP-6/TP-13). (**C**) Colony diameters of the metalaxyl-resistant strain PSM7-3, non-edited control transformants (TM-1/TM-3), and PsALG8 knockout transformants (TM-5/TM-6). Error bars represent the standard deviation (SD) of three biological replicates. ** *p* < 0.01 compared with the corresponding wild-type control (Student’s *t*-test).

We measured the mycelial growth of four PsALG8 knockout transformants, as well as the wild-type isolate and non-edited transformants. After incubation at 25 °C for 7 days, the mycelia growth of TP-6 and TP-13 was significantly reduced in comparison with wild-type isolate P7076 and the non-knocked isolates (TP-1 and TP-2) (*p* < 0.01). Colony diameter was measured as an indicator of mycelial growth. The average colony diameter of P7076 and the non-knocked isolates was 4.03 cm, while the average diameter of TP-6 and TP-13 was only about 3.51 cm (Figure 5). Similar results were observed in the resistant mutant PSM7-3 and its transformants (TM-5 and TM-6). The average colony diameter of PSM7-3 and the non-knocked isolates (TM-1 and TM-3) was 4.05 cm, whereas the average diameter of TM-5 and TM-6 was 3.62 cm (Figure 5A,C), showing a significantly decreased growth ability compared with the wild-type PSM7-3 (*p* < 0.01). These results indicate an important role of PsALG8 in mycelial growth.

## 4. Discussion

Plant diseases caused by *Phytophthora* pathogens represent a major threat to global agricultural and horticultural production. Oomyceticides, such as metalaxyl, have traditionally displayed excellent performance in controlling *Phytophthora* diseases. However, the rapidly evolving resistance of *Phytophthora* to these chemicals significantly undermines their efficacy. This challenge is particularly concerning in high-value woody crops, such as walnut (*Juglans* spp.), where *Phytophthora* infections frequently lead to severe collar rot, tree decline, and mortality. Given that chemical intervention remains a cornerstone for managing these pathogens, there is an urgent need to elucidate the genetic basis of resistance to ensure sustainable crop protection. Many of the *Phytophthora* species we isolated lack publicly available whole-genome sequences—for example, the uncharacterized *Phytophthora* species currently responsible for extensive walnut (*Juglans* spp.) mortality in Chongqing, China—so, in this study, we utilized *P. sojae* as a model system to investigate the molecular mechanisms of metalaxyl resistance, providing a foundation for broader applications in oomycete management.

Following prolonged intensive application, metalaxyl resistance has been recurrently detected in diverse oomycetes, such as Phytophthora infestans [15], *P. viticola* [9,43], and *Pythium* spp. [13,44,45]. However, metalaxyl resistance in *P. sojae* has not been reported in natural populations to date. We speculated that the resistance risk and the corresponding resistance mechanism of *P. sojae* to metalaxyl may be different from that of other oomycete species. In this study, we successfully generated six stable resistant mutants after 30 times of repeatedly exposing *P. sojae* to metalaxyl. All the mutants exhibited a high level of metalaxyl resistance (RF > 2000). In terms of the generation of high resistance, after a short time of exposure, we suggest that *P. sojae*, similar to *P. infestans*, is also at high risk to develop resistance to metalaxyl. Therefore, combinations and rotations of metalaxyl with other oomyceticides are recommended for the effective control of *P. sojae*.

Molecular mechanism of metalaxyl resistance still remains unknown. Previous studies have revealed that metalaxyl inhibits the synthesis of ribosomal RNA, suggesting that it may target RNA polymerase I. Randall et al. investigated the RPA190 gene, which encodes the largest subunit of RNA polymerase I, and found an SNP (causing an amino acid change in F382Y) conferring metalaxyl insensitivity in *P. infestans* [11]. However, using both DNA sequencing and high-resolution melt assays to distinguish RPA190 alleles, Michael et al. found that the F382Y was absent from certain resistant isolates of *P. infestans* from Europe, Mexico, and North America [12]. Therefore, it is likely that other genes contribute to metalaxyl resistance. No nonsynonymous mutations were detected in the coding regions of genes encoding RNA polymerase I subunits in the six resistant mutants examined in this study. Therefore, coding-sequence mutations in RNA polymerase I subunits are unlikely to explain resistance in these mutants; however, other mechanisms affecting RNA polymerase I function cannot be excluded.

The most common molecular mechanism of fungicide resistance is point mutations in target genes [11,37,38,39,40,46,47]. Thus, to detect genes associated with metalaxyl resistance, we performed high-throughput genome re-sequencing analysis of the wild-type parental isolate and the six metalaxyl-resistant laboratory-induced strains. A total of 324 genes with point mutations were detected in at least one metalaxyl-resistant isolate. We further investigated the conservation and divergence of the 324 genes across the six metalaxyl-resistant laboratory-induced strains. Most of these genes are unique to each metalaxyl-resistant laboratory-induced strain. However, there are 69 genes common to all the metalaxyl-resistant isolates. Considering that all laboratory-induced resistant mutants were derived from the same wild-type isolate, we narrowed down to these 69 genes and considered them as potential targets associated with metalaxyl resistance. High resistance level are mainly caused by homozygous mutations, whereas heterozygous mutations mainly confer moderate resistance level [37,46,47,48]. Therefore, we searched for the most frequently observed homozygous point mutations in the six metalaxyl-resistant isolates. Finally, we identified PsALG8 (EGZ16653), which has the most frequently observed homozygous SNP G1264C (V422L). The identity between PsALG8 and the homologous protein of Saccharomyces cerevisiae is 29.9%, and the similarity is 46.5%. Sequence analysis found PsALG8 includes ten transmembrane domains and an Alg6_Alg8 domain. The point mutation V422L occurs near the ninth transmembrane domain. Multiple sequence alignments revealed that the valine at position 422 is conserved in all the tested 11 oomycete species (e.g., *P. sojae*, *P. infestans*, *P. ramorum*, *P. capsici*, *Pythium ultimum*, and so on). Collectively, These results suggest that the V422L substitution may be associated with laboratory-induced metalaxyl resistance, although further genetic validation is required. Nevertheless, it will be of great significance to investigate how PsALG8 modulates mycelial growth and metalaxyl susceptibility, how the V422L substitution drives metalaxyl resistance, and whether ALG8 homologs from other Phytophthora species contribute to fungicide resistance. In particular, targeted introduction of the PsALG8 V422L mutation is required in future work to verify whether this single substitution alone is sufficient to confer metalaxyl resistance. Because a non-selected P7076 control subjected to the same number of transfers was not included in the original design, mutations arising from repeated subculturing cannot be excluded. Therefore, the genome re-sequencing results were used to identify candidate mutations associated with laboratory-induced metalaxyl resistance rather than to establish definitive causality.

Eukaryotic protein N-glycosylation involves the transfer of an oligosaccharide from lipid-linked oligosaccharides (LLOs) to asparagine in acceptor proteins [49]. The biosynthesis of LLOs takes place in the luminal side of the endoplasmic reticulum (ER) and requires glucose as a donor substrate. ALG8 catalyzes the second glucose residue to LLOs [50]. In fact, in addition to the above biochemical function in N-glycosylation, much remains unknown about ALG8. Mutations in human ALG8 have been implicated in the congenital disorder of glycosylation CDG-Ih [51]. In *Saccharomyces cerevisiae*, ALG8 is not essential for vegetative growth [50]. Currently, only a limited number of oomycete genes have been functionally characterized via the CRISPR/Cas9 system. In this study, we used the CRISPR/Cas9 system to knock out PsALG8 in *P. sojae* to investigate its biological functions [52,53,54,55,56,57,58,59,60,61,62,63,64,65,66]. In this study, we used the CRISPR/Cas9 system to knock out PsALG8 in *P. sojae* to investigate its biological functions. Our results showed that deletion of PsALG8 reduced mycelial growth compared with the parental isolate and non-edited transformants. Distinct phenotypic changes after ALG8 knockout in yeast vs. *P. sojae* suggest interspecific functional divergence of this gene. Notably, loss of *PsALG8* improves metalaxyl susceptibility but hardly affects pathogen virulence (Figure S3) [35]. In summary, our results suggest that PsALG8 is involved in metalaxyl sensitivity and mycelial growth in *P. sojae* under laboratory conditions. However, because the resistant strains were generated by laboratory selection and because precise allelic replacement was not performed, the present data do not demonstrate that the V422L substitution alone confers metalaxyl resistance. Further studies using non-selected passage controls, complementation, allelic replacement, and naturally occurring field isolates are required to determine the role of PsALG8 variation in field-relevant resistance. The resistant mutants analyzed in this study were generated under repeated metalaxyl selection in vitro. Therefore, the mechanisms identified here may not fully represent those operating in natural field populations. Field population monitoring will be required to determine whether similar mutations occur under agricultural conditions. Because the resistant mutants were generated in vitro, open-field inoculation experiments were not conducted for biosafety reasons. Instead, pathogenicity and fitness were evaluated under controlled conditions. Future field population monitoring and sensitivity surveys will be necessary to assess the practical risk of metalaxyl resistance in natural *P. sojae* populations.

**Figure 6:**
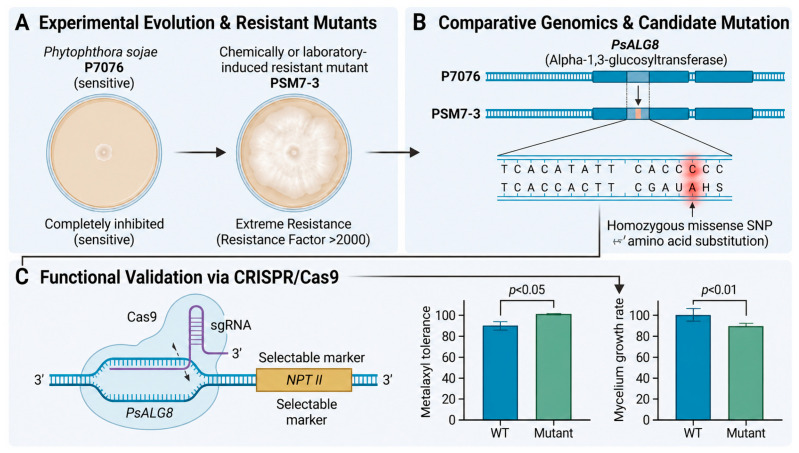
**Graphical summary of this study.** (**A**) Sensitivity of the parental isolates P7076 and metalaxyl-resistant mutant PSM7-3 to metalaxyl. (**B**) Identification of candidate genes associated with metalaxyl resistance. (**C**) Candidate gene *PsALG8* involved in metalaxyl tolerance and mycelial growth in *P. sojae*.

As far as we know, this study is the first to systematically assess the resistance risk and mechanism of *P. sojae* to metalaxyl. PsALG8 is predicted to encode an ALG8-family α-1,3-glucosyltransferase. While previous studies have identified homologs of ALG8 in oomycetes, this work is the first to investigate its biological functions in regulating mycelial growth and metalaxyl sensitivity in *Phytophthora*. However, the enzymatic activity and downstream targets of PsALG8 were not experimentally characterized in this study. We demonstrated that *P. sojae* is at high risk of developing resistance to metalaxyl. Meanwhile, we provide a reliable set of candidate genes for further investigating the molecular mechanism of metalaxyl resistance in oomycetes. Overall, this study identifies PsALG8 as a candidate gene associated with metalaxyl sensitivity and mycelial growth in laboratory-induced resistant strains of *P. sojae* (Figure 6). Further work is required to determine whether PsALG8 variation contributes to metalaxyl resistance in natural populations and to establish the causal role of the V422L substitution. These findings not only provide a reliable gene set for further investigating the molecular mechanism of metalaxyl resistance in oomycetes but also offer potential targets for developing novel resistance management strategies. Such insights are crucial for safeguarding diverse crops, including walnut orchards, against the escalating threat of *Phytophthora* diseases.

## Data Availability

The original contributions presented in this study are included in the article/Supplementary Material. Further inquiries can be directed to the corresponding authors.

## Supplementary Materials

The following supporting information can be downloaded at: https://www.mdpi.com/article/10.3390/jof12070479/s1. Figure S1: Classification of SNPs discovered in *P. sojae* re-sequencing metalaxyl-resistant mutants A. Percentage distribution of all base substitution types among detected SNPs; the G→A (25.22%) and C→T (23.29%) transitions represent the two most abundant mutation types. B. Genomic location proportion of total SNPs: 29.35% in coding sequences (CDS), 7.38% in introns, and 63.27% in intergenic regions; Figure S2: Generation and identification of PsALG8 mutants by CRISPR/Cas9 deletions A. CRISPR/Cas9-mediated *PsALG8* knockout model, and primer pair location for varification of *PsALG8* knockout transformants. *PsALG8* was replaced with NPT II. Red arrow indicates the sgRNAs cleavage site. F2R2 is used to verify whether the gene has been knocked out, and F1R1 is used to verify whether the knocked-out gene is replaced by NPT II. B. *PsALG8* knockout transformants TP-6 and TP-13 were generated in the wild-type background (P7076). C. *PsALG8* knockout transformants TM-5 and TM-6 were obtained in the metalaxyl-resistant mutant background (DM7-3); Figure S3. Pathogenicity of transformants in the wild-type background (P7076). A. Lesion symptoms on soybean hypocotyls inoculated with the wild-type isolate P7076, PsALG8 knockout transformants TP-6 and TP-13, and the non-edited control transformant TP-2. B. Quantification of lesion lengths corresponding to panel A. Data are presented as mean ± standard deviation from three independent biological replicates. Values with the same lowercase letter are not significantly different at *p* < 0.05 level according to Duncan’s multiple range test; Table S1: All primers used in this study; Table S2: Read statistics of *P. sojae* genome sequencing; Table S3: Candidate genes associated with metalaxyl resistance; Table S4: ID numbers of PsAGL8 homologous proteins; Table S5: P7076 Candidate resistance genes of acquired P7076 metalaxyl resistance strains.
