# Supplementary figures and images for: Genome Re-Sequencing and Functional Analysis Reveal an α-1,3-Glucosyltransferase Conferring Metalaxyl Resistance in Phytophthora sojae
Source: J Fungi (Basel). 2026 Jun 30;12(7):479. doi: 10.3390/jof12070479 (PMC13412751; doi:10.3390/jof12070479)

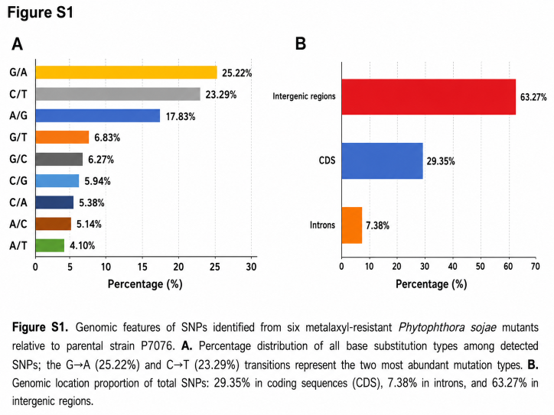

Supplement: Supplementary file 1 [file jof-12-00479-s001.zip › jof-4334382-supplementary/Figure S1.tif]

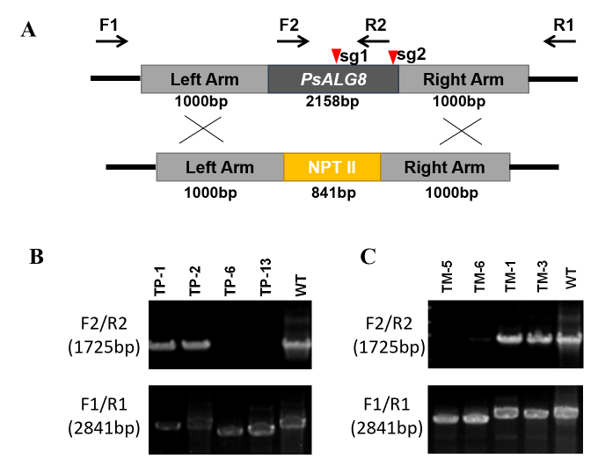

Supplement: Supplementary file 1 [file jof-12-00479-s001.zip › jof-4334382-supplementary/Figure S2.tif]

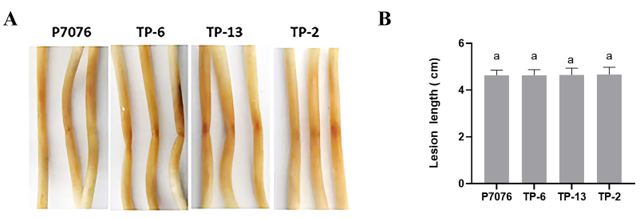

Supplement: Supplementary file 1 [file jof-12-00479-s001.zip › jof-4334382-supplementary/Figure S3.tif]
